# *In vitro* studies of H520 cell cycle and apoptosis by anlotinib combined with radiotherapy

**DOI:** 10.1111/1759-7714.13780

**Published:** 2021-01-12

**Authors:** Lu Guo, Luyao Zhang, Yan Guan, Yan Li, Chufeng Zhang, Qisen Guo

**Affiliations:** ^1^ Shandong Cancer Hospital and Institute, Shandong University Shandong First Medical University, Shandong Academy of Medical Sciences Jinan China; ^2^ Oncology Affiliated Hospital of Weifang Medical University Weifang China; ^3^ Breast and Thyroid Surgery Affiliated Hospital of Weifang Medical University Weifang China

**Keywords:** Anlotinib, cell cycle/apoptosis, lung cancer, radiotherapy

## Abstract

**Background:**

To study in vitro the effects of anlotinib combined with radiotherapy on the lung cancer H520 cell cycle and apoptosis.

**Methods:**

The log growth period H520 cells were divided into the control group, anlotinib group (A group), radiotherapy group (RT group) and combined group (A + RT group). Cell cycle and apoptosis were detected by flow cytometry and changes in H520 cell cycle and apoptosis were analyzed in each group.

**Results:**

Anlotinib was determined to significantly inhibit cell growth in all groups, both alone, or in combination with radiotherapy. After receiving corresponding treatments, the proportions of G2/M‐phase cells in the control group, A group, RT group and A + RT group were different, and statistically significant (F = 32.086, *P* < 0.001). The apoptotic cell statistics of H520 cells in the control group, A group, RT group and A + RT group were significantly different (F = 44.537, *P* < 0.01). The relative expression of CDK1 in each group of cells was 0.04 ± 0.02, 0.07 ± 0.12, 0.81 ± 0.11, and 0.56 ± 0.16, respectively. There were differences between the groups by analysis of variance which were statistically significant (F = 58.36, *P* < 0.0001). The relative expression of cycle B in each group of cells was 0.27 ± 0.05, 0.40 ± 0.16, 0.65 ± 0.14, and 0.57 ± 0.13, respectively. There were differences between the groups by analysis of variance which were statistically significant (F = 10.77, *P* = 0.0002).

**Conclusions:**

Anlotinib had an inhibitory effect on lung cancer H520 cell proliferation. A higher rate of apoptosis and G2/M phase block was observed in the anlotinib‐radiotherapy combined group. Anlotinib combined with radiotherapy was able to synergistically inhibit tumor cell growth.

**Key points:**

Anrotinib combined with radiotherapy can synergistically inhibit tumor cell growth.

## Graphical abstract

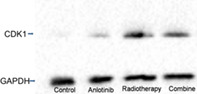

Anlotinib had an inhibitory effect on lung cancer H520 cell proliferation. Anlotinib combined with radiotherapy was able to synergistically inhibit tumor cell growth.

## Introduction

Lung cancer has the highest incidence and mortality rate of malignant tumors worldwide, and the five‐year survival rate of patients with advanced lung cancer is still less than 15%.^1^ Non‐small cell lung cancer (NSCLC) accounts for 80% of lung cancers with no specific clinical signs and symptoms in the early stages. Squamous cell lung cancer (SqCLC) is a unique type of NSCLC, accounting for approximately 25%–30% of all NSCLC cases.^2^ Compared to nonsquamous NSCLC, SqCLC has no safe and effective molecularly targeted agents for clinical use, and its treatment faces greater challenges. Anlotinib is a new small molecule multitargeted tyrosine kinase inhibitor developed by China's Chia Tai Tianqing Pharmaceutical Group, which can effectively inhibit VEGFR, PDGFR, FGFR, c‐Kit, Met and other kinases, and has the functions of antitumor angiogenesis and tumor growth inhibition.^3^, ^4^ In addition to antiangiogenesis, it is unknown whether anlotinib affects cell cycle and apoptosis. The aim of this study was mainly to observe the effect in vitro of anlotinib combined with radiotherapy on cell cycle and apoptosis in lung squamous carcinoma cells.

## Methods

### Materials

Anlotinib (effective dose 12 mg/tablet, Chia Tai Tianqing Pharmaceutical Group Co., Ltd); DMEM high glucose medium (Gibco, USA); fetal bovine serum (HyClone, USA); apoptosis detection kit (Becton, Dickinson and Company, USA); cell cycle analysis kit (Beyotime Biotechnology Co., Ltd. China); human lung cancer H520, H226 and H2170 cell lines (provided by the Central Laboratory of Shandong Cancer Hospital); and linear accelerator (Elekta AB, Sweden).

#### Cell culture

The human lung squamous carcinoma cell lines were inoculated in 25 cm^2^ culture flasks with DMEM medium containing 10% fetal bovine serum and incubated at 37°C with 5% CO_2_ in an incubator. The cells were observed daily under an inverted microscope and passaged every 2–3 days. Logarithmic growth period cells were taken for experiments.

#### Cell irradiation

The radiotherapy group and combined group were irradiated by ^60^Coγ linear accelerator, source to surface distance = 100 cm, radiation field 40 cm × 40  cm, irradiation dose 2 Gy, and the irradiated cells were placed in a 37°C with 5% CO_2_ incubator for further culture.

#### MTT method detection of proliferation inhibitory effect on lung cancer cells by each treatment group

Logarithmic cells were used to make a cell suspension of 5 × 10^4^ cells/mL and they were then inoculated in a 96‐well cell culture plate. Blank control groups were set up with six double wells in each group. After 72 hours incubation at 37°C and 5% CO_2_ in an incubator, 20 uL MTT was added to each well and incubated for another four hours, the supernatant was discarded, 150 uL DMSO was added, and the crystalline pellet was fully dissolved by shaking for 15 minutes at room temperature. The absorbance value of each well was measured at 490 nm, and cell growth inhibition rate was calculated. Cell growth inhibition rate = (1 − treatment group D [490 nm]/control group D [490 nm]) × 100%.

#### Flow cytometry detection of cell cycle and apoptosis

Logarithmic cells were taken and 2 mL of cell suspension at a concentration of 5 × 10^4^ cells/mL was aspirated and added to a 35 mm culture dish. After incubation in DMEM medium for 12 hours(ie, cell wall growth), the cells were divided into the control group, A group, RT group, and A + RT group. Anlotinib 25% half inhibitory concentration: 120 ng/L was selected as the experimental drug concentration. Anlotinib (120 ng/L) was added to the A group and the A + RT group, with three parallel samples set up for each group. After 24 hours of incubation, 2 Gy irradiation was applied to the radiotherapy group and combined groups, and incubation was terminated 24 hours after irradiation. The cells in each group were routinely digested, centrifuged and resuspended. Prechilled PBS was used to wash the resuspended cells which were then fixed in 70% alcohol at 4°C and left overnight. The next day, the precipitated cells were again centrifuged, the fixative discarded, and 0.5 mL of the prepared pyridine iodide staining solution added to each tube of cells in order to slowly and fully resuspend the cell precipitate. Exposure to light was avoided and they were placed in a warm bath at 37°C for 30 minutes, and the cell cycle detected by flow cytometry. Apoptosis detection was as follows: After routine digestion, centrifugation and resuspension of cells with PBS, the cells were again centrifuged, the PBS aspirated and about 100 μL binding buffer added to each tube and the cells were then resuspended. Then 7‐AAD (5 μL) and PE (5 μL) was added, respectively, they were incubated for 15 minutes at room temperature without light, and 400 μL binding buffer added, re‐suspended with a pipet, and apoptosis detected by flow cytometry. All the above experiments were repeated three times.

#### Western‐blot detection of CDK1, cycle B and caspase‐3 expression in cell lines

Extraction of total protein: The culture medium was discarded and the cells rinsed with PBS. The cell lines were then lysed at a ratio of 150–250 μL of lysis solution per well in a six‐well plate, and transfered to an EP tube. They were placed on ice for 30 minutes and then shaken for 15 seconds every five minutes before being centrifuged at 12000 rpm/minute for 10 minutes at 4°C, and the supernatant taken as total cell protein.

Western‐blot procedures: The protein solution was added into the same volume of buffer and boiled at 100°C for 10 minutes, then frozen on ice. The separation gel was prepared, the sample added and electrophoresis performed at 80–100 V. The membrane was then transferred on ice at 90 V for 90 minutes, sealed with 5% skimmed milk powder for two hours and the sealing solution was diluted with primary antibody at 4°C overnight. The membrane was washed with PBTS, a secondary antibody added and incubated for two hours. The membrane was then washed again and exposed with an imaging system. The images were analyzed by image processing Software Image Laboratory and plotted using Graph Pad Prism 8.0.

### Statistical analysis

SPSS19.0 software was used for statistical analysis, and the experimental results were expressed as^−^x ± s. One‐factor variance analysis was used for mean value comparison between multiple groups, and the Holm‐Sidak method was used for comparison between results within groups. *P* < 0.05 was considered statistically significant.

## Results

### Inhibitory effect of anlotinib on cells

In the anlotinib group, compared with the control group, the inhibition rate of H520 cell line was 1.87 ± 1.33% which was significantly higher than that in the control group (t = 3.4440, *P* = 0.0063). The inhibition rate of H226 cell line was 2.01 ± 1.17% which was significantly higher than that in the control group (t = 4.2081, *P* = 0.0018). The inhibition rate of H2170 cell line was 1.56 ± 1.03% which was significantly higher than that in the control group (t = 3.7099, *P* = 0.0040).

**Figure 1:**
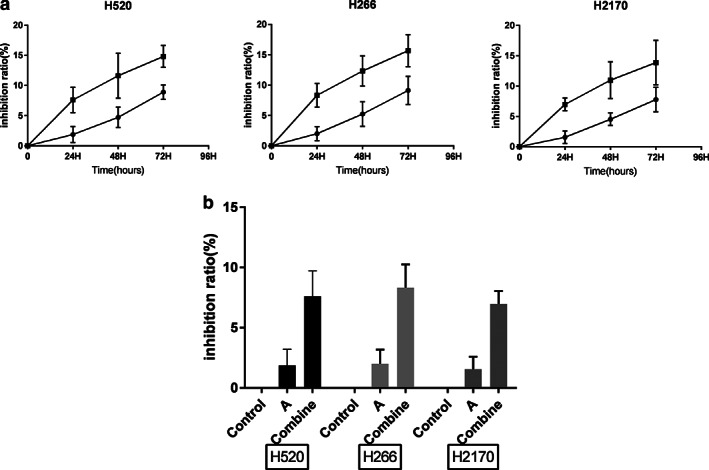
(**a**) Inhibitory effect of anlotinib on H520, H226 and H2170 cells (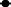) Anlotinib, (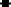) Combined, (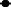) Anlotinib, (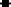) Combined, (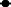) Anlotinib, (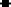) Combined. (**b**) Inhibitory effect of anlotinib on H520, H226 and H2170 cells (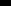) H520, (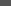) H226, (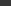) H2170.

In the combined group, compared with the control group, the inhibition rate of H520 cell line was 7.61 ± 2.11% which was significantly higher than that in the control group (t = 5.6371, *P* = 0.0002). The inhibition rate of H266 cell line was 7.61 ± 2.11% which was significantly higher than that in the control group (t = 6.8225, *P* = 0.0000).The inhibition rate of H2170 cell line was 7.61 ± 2.11% which was significantly higher than that in the control group (t = 8.9825, *P* = 0.0000) (Fig 1).

After receiving anlotinib and anlotinib combined radiotherapy for lung squamous cell lines H520, H266 and H2170, respectively, the comparison and analysis of cell proliferation before and after showed that all lung squamous cell lines in each group were inhibited by anlotinib, and the inhibitory effect was more significant when combined with radiotherapy.

In order to select the appropriate drug concentration and action time, we selected the appropriate drug concentration for this study by selecting the appropriate action time based on the concentration‐inhibition rate graph of the H520 cell line after receiving anlotinib, radiotherapy and combined intervention, and by comparing the cell inhibition under different concentrations.

**Figure 2:**
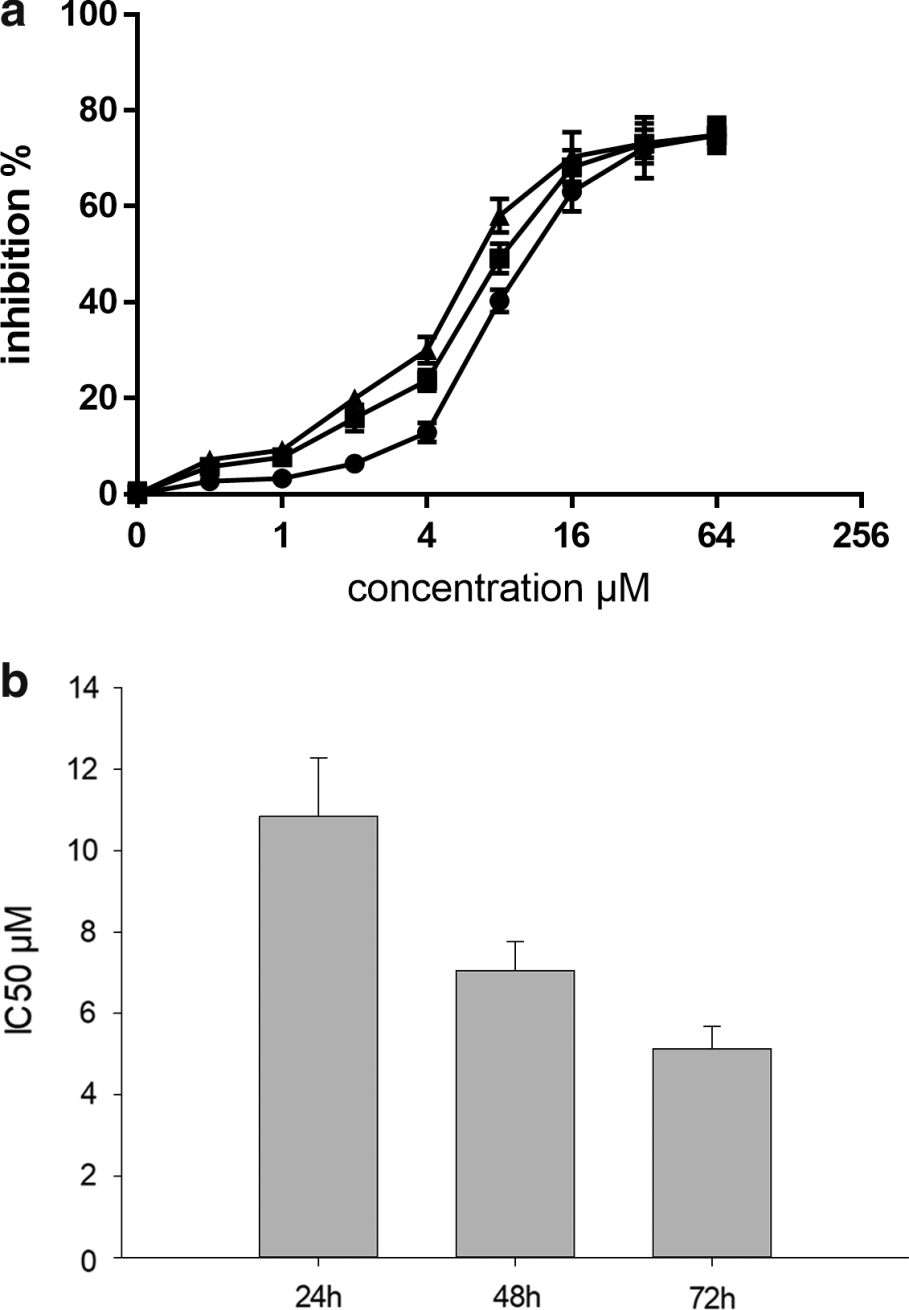
(**a**) Inhibitory effect of anlotinib on H520 cells (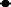) 24 hours, (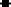) 48 hours, (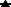) 72 hours. (**b**) Inhibitory effect of anlotinib on H520 cells.

After gradient concentrations of anlotinib were applied to the H520 cell lines for 24, 48, and 72 hours, the cell inhibition rate was calculated based on the absorbance values, and the average of the six‐well cell inhibition rate was used as the average inhibition rate for the corresponding concentrations to plot the concentration‐inhibition rate for the three time periods. The three time periods calculated for the IC50 were 10.84, 7.056, and 5.135 μM respectively, and analysis of variance showed that there was a difference between them (F = 78.866, *P* < 0.001), with pairwise comparison suggesting that the difference between them was statistically significant. Anlotinib action for 24 hours corresponded to IC50 = 10.84 μM with a positive inhibitory effect on cell proliferation (Fig 2).

After 24 hours of action of gradient concentrations of anlotinib on H520, there was a significant difference in the inhibition rate of H520 cell proliferation by analysis of variance (F = 618.398, *P* < 0.01). When pairwise compared with the control group, the difference in the proliferation inhibition rate of H520 cells at a concentration of 2 μM began to be statistically significant (t = 3.507, *P* = 0.007), while the difference between 0.5 and 1 μM on the proliferation inhibition rate of H520 cells was not significant (t = 1.490, *P* = 0.371; t = 1.825, *P* = 0.322). Therefore, 2 μM was chosen as the test drug concentration.

### Cell cycle distribution

**Figure 3:**
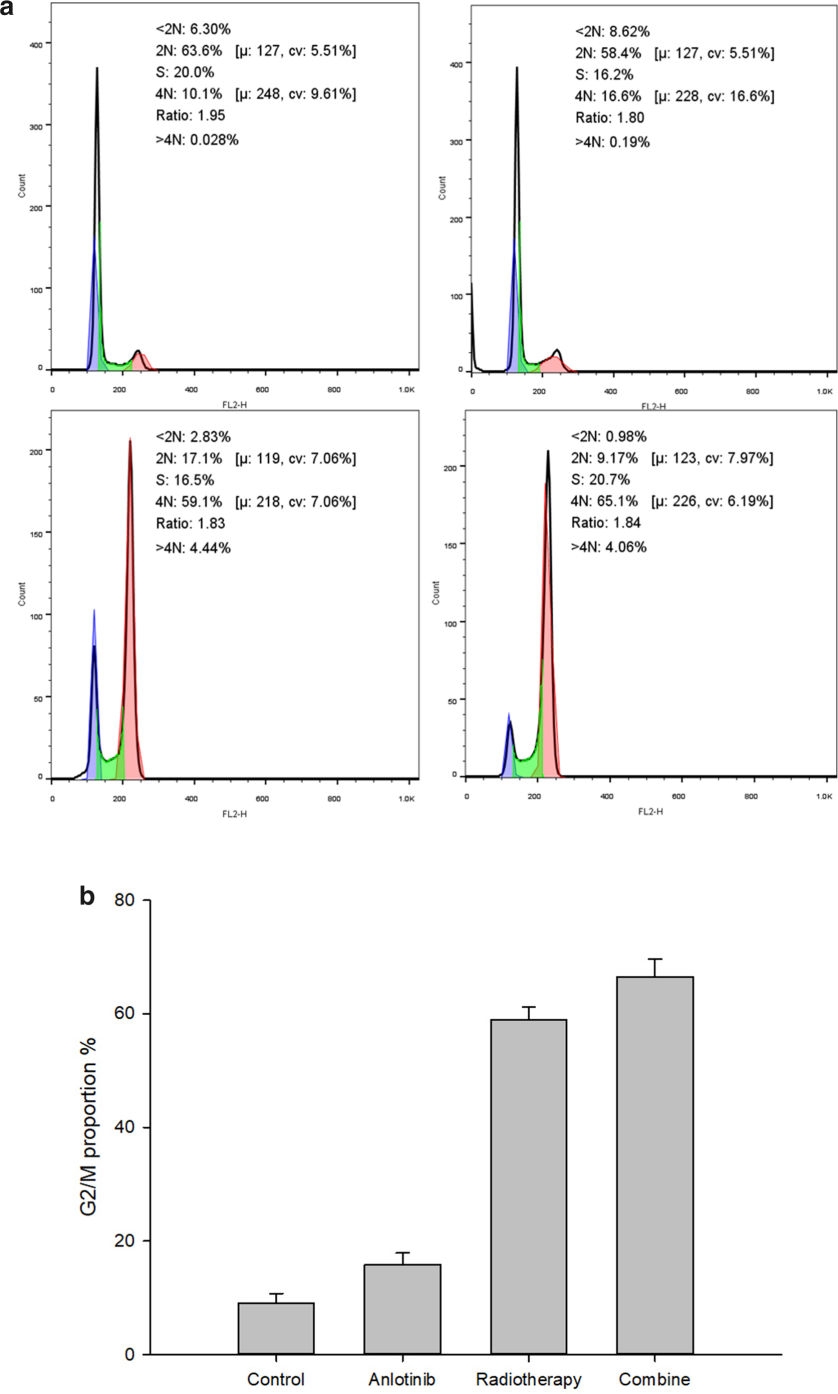
(**a**) Flow cytometric results of four groups. (**b**) Comparison of G2/M proportion in four groups.

After receiving the corresponding treatments, there was a difference in H520 cell cycle distribution between the control group, A group, RT group, and A + RT group (F = 950.284, *P* < 0.001). The proportion of G2/M‐phase cells in the control group was (9.01 ± 1.73%), A group (15.81 ± 2.08%), RT group (58.91 ± 2.32%), and A + RT group (66.56 ± 3.01%). Pairwise comparisons suggested that the A + RT group was higher than RT group (t = 5.681, *P* < 0.001), RT group was higher than the A group with significant difference (t = 32.005, *P* < 0.001), and that A group was higher than the control group (t = 5.050, *P* < 0.001) (Fig 3).

### Cell apoptosis rate

**Figure 4:**
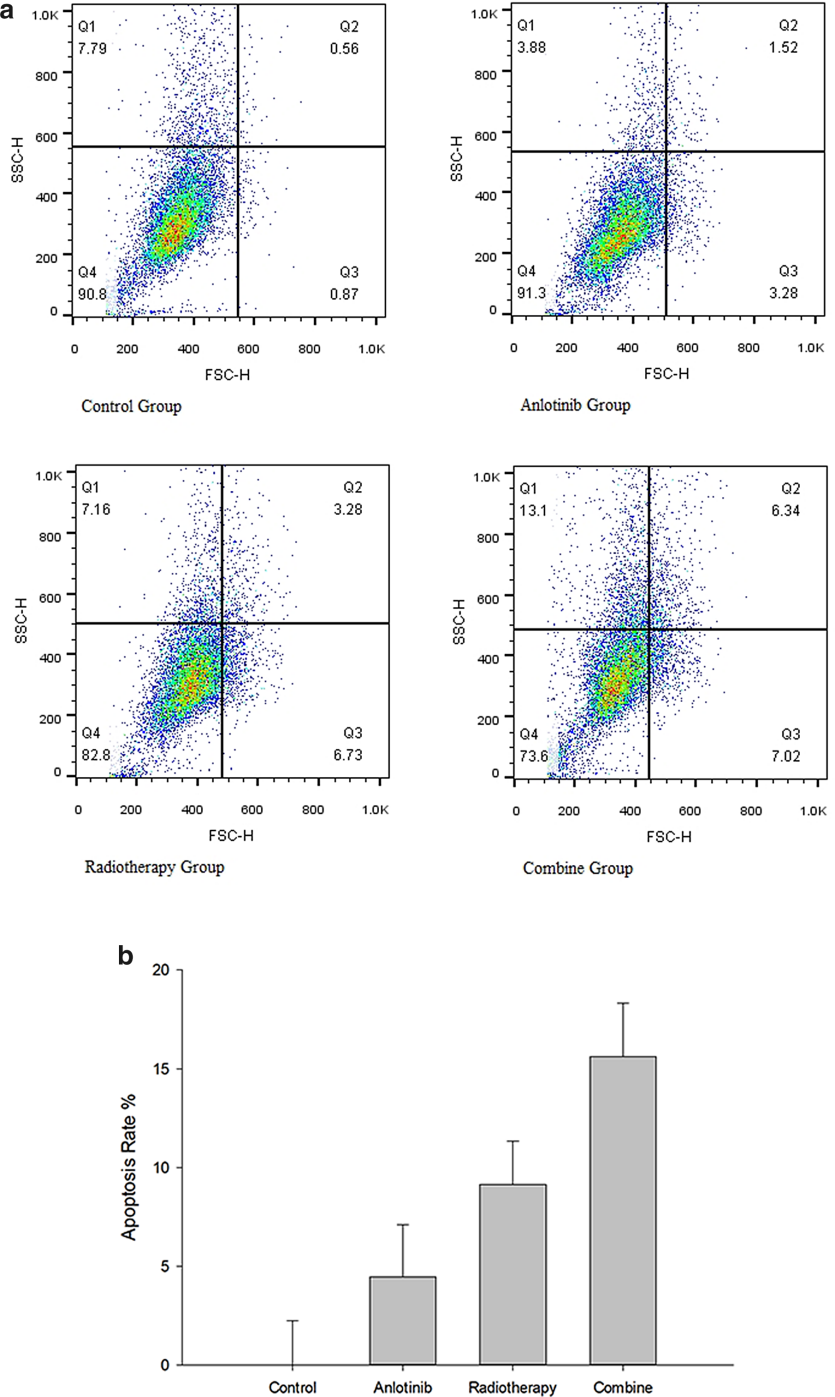
(**a**) Apoptosis of cell lines in four groups. (**b**) Apoptosis of cell lines in four groups.

According to the statistics of H520 apoptotic cells in the control group, A group, RT group and A + RT group by flow cytometry, the apoptotic rates of H520 cells in each group were 0 ± 2.23%, 4.47 ± 2.63%, 9.15 ± 2.19% and 15.61 ± 2.71%, respectively. Analysis of variance showed that there were significant differences (F = 44.537, *P* < 0.01). Pairwise comparisons between groups showed that the apoptosis rate was higher in the A + RT group than in the RT group (t = 4.565, *P* < 0.001), in the RT group than in A group (t = 3.307, *P* = 0.007), and in the A group than in the control group (t = 3.159, *P* = 0.005), all with statistically significant differences (Fig 4).

### Expression of CDK1 and cycle B in cell lines

The relative expression of CDK1 in each group of cells was 0.04 ± 0.02, 0.07 ± 0.12, 0.81 ± 0.11, and 0.56 ± 0.16, respectively. There were differences between the groups in analysis of variance, which were statistically significant (F = 58.36, *P* < 0.0001).

**Figure 5:**
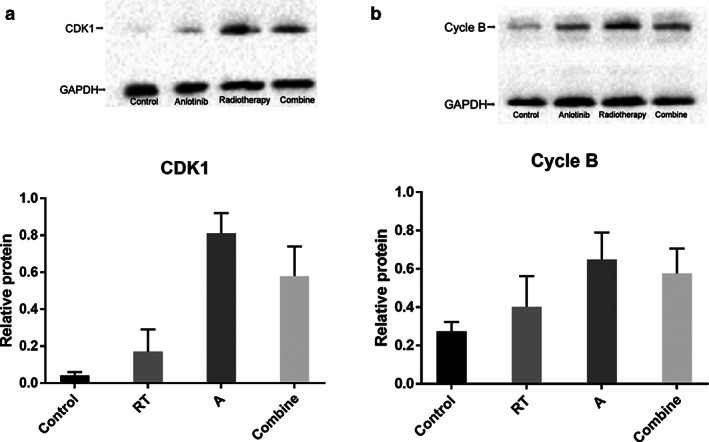
(**a**) Expression and statistical analysis of CDK1 in each group of cells. (**b**) Expression and statistical analysis of CDK1 in each group of cells (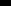) Control, (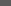) RT, (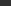) A, (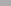) Combined. (**c**) Expression and statistical analysis of cycle B in each group of cells. (**d**) Expression and statistical analysis of cycle B in each group of cells (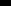) Control, (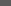) RT, (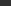) A, (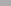) Combined.

The relative expression of CDK1 in each group of cells was 0.27 ± 0.05, 0.40 ± 0.16, 0.65 ± 0.14, and 0.57 ± 0.13, respectively. There were differences between the groups in analysis of variance, which were statistically significant (F = 10.77, *P* = 0.0002) (Fig 5).

### Expression of caspase‐3 in cell lines

**Figure 6:**
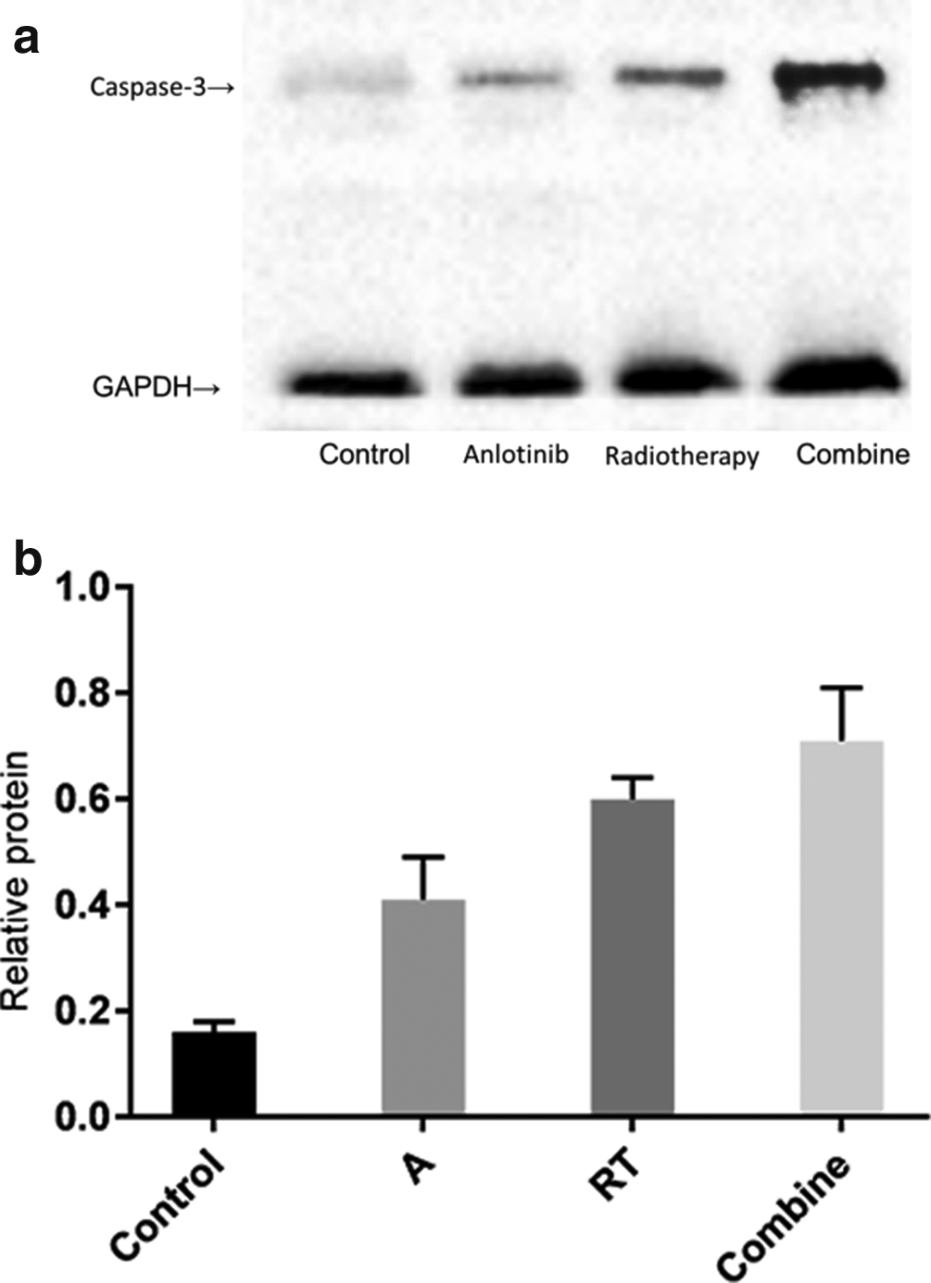
(**a**) Expression and statistical analysis of caspase‐3 in each group of cells. (**b**) Expression and statistical analysis of caspase‐3 in each group of cells (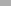) Combined, (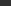) RT, (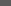) A, (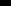) Control.

The relative expression of caspase‐3 in the control group, A group, RT group, and combined group was 0.16 ± 0.02, 0.41 ± 0.08, 0.60 ± 0.0.04, and 0.72 ± 0.10, respectively. There were differences between the groups in analysis of variance, which were statistically significant (F = 75.74, *P* < 0.0001). Comparison between groups showed that the apoptosis rate in the combined group was higher than that in the A and RT groups (q = 7.661, *P* < 0.0001; q = 2.809, *P* = 0.0284), and that the difference was statistically significant (Fig 6).

## Discussion

Lung cancer is one of the most common malignant tumors threatening the whole of mankind. Its morbidity and mortality rates rank first among all malignant tumors, and the number of lung cancer cases in China is increasing rapidly. Of the total number of lung cancers, 80%–85% are non‐small cell lung cancer (NSCLC), of which 65%–80% of NSCLC patients are already at an advanced stage at the time of diagnosis and have a low rate of surgical resection.^5^ Squamous cell lung cancer (SqCLC) accounts for about 25%–30% of all NSCLC, and this pathology is often treated with platinum‐containing combination chemotherapy, with an overall remission rate of less than 50%.^6^ Since there are currently no targeted drugs that specifically target SqCLC targets in the clinic, the overall remission rate of SqCLC remains to be improved.

Anlotinib is a novel small molecule, multitargeted tyrosine kinase inhibitor that is currently included in clinical trials for a variety of cancers. Its targets of action include significant inhibitory activity against angiogenesis‐related kinases such as VEGFR1/2/3, FGFR1/2/3 and other tumor‐associated kinases such as PDGFR, c‐Kit and Ret that are associated with cell proliferation; other partial kinase targets under malignant investigation include Aurora‐B, c‐FMS, and DDR1, and partial kinase mutants, such as *PDGFR*, *c‐Kit*, *Met*, and *EGFR*, and the inhibitory activity of anlotinib against partial mutants has been found to be even stronger than that of wild‐type.^7^, ^8^, ^9^ The probability of *EGFR*‐sensitive mutations in Chinese patients with advanced lung adenocarcinoma is about 50%. On one hand, antiangiogenic drugs target specific monoclonal antibodies against VEGFR, such as bevacizumab and cetuximab, which have high target specificity and require genetic testing before application, and on the other, they inhibit the VEGFR domain of small‐molecule tyrosine kinase inhibitors, such as apatinib and sorafenib, which are multitargeted drugs.^10^, ^11^ There are no monoclonal targeting agents that specifically target SqCLC targets. Clinical remission has been reported in some patients after third‐line application of tyrosine kinase inhibitors, with significantly fewer side effects than chemotherapy.

A number of clinical trials have shown that the combination of anlotinib and radiotherapy is effective in the treatment of advanced lung squamous cell carcinoma, but there are still some patients with radiation resistance. Therefore, it is of great significance to investigate the effect of anlotinib combined with radiotherapy on apoptosis and the mechanism of radiosensitization in squamous cell carcinoma of lung cells in vitro. This study assesses the efficacy of the treatment by combination radiotherapy. Previous literature has reported that the IC_50_ for anlotinib is about 10 μM, which is very similar to the IC_50_ calculated by drug concentration and cell inhibition rate in the present study.^12^ Since combination radiotherapy acted on the H520 cell line in this study, in order to effectively observe the inhibitory effect of anlotinib and radiotherapy on cell proliferation, lower drug concentrations were used in the experiment to compare the effect of gradient concentrations on the cell inhibition rate, and there was no significant difference between the inhibitory effect of 0.5 and 1 μM and the control group (t = 1.490, *P* = 0.371; t = 1.825, *P* = 0.322). Effective inhibition of cell proliferation started from 2 μM (t = 3.507, *P* = 0.007), so this concentration was used as the working concentration in this study. The results confirmed that the H520 cell apoptosis rate was higher in the combined group than in the radiotherapy group (t = 4.565, *P* < 0.001), higher in the radiotherapy group than in the anlotinib group (t = 3.307, *P* = 0.007), and higher in the anlotinib group than in the control group (t = 3.159, *P* = 0.005), indicating that the combination of anlotinib and radiotherapy could inhibit the proliferation of H520 cells, and the treatment protocol containing radiotherapy had a stronger apoptosis‐inducing effect on the cells than anlotinib alone. Another clinical implication of this finding is that the systemic therapeutic effect of anlotinib cannot substitute for the therapeutic effect of radiotherapy in local treatment. This result has also been confirmed by numerous clinical trials.^13^, ^14^, ^15^

Different antiangiogenic drugs have different cell cycle blockades. Studies in gastrointestinal tumors and hepatocellular carcinoma have shown that sorafenib, BD0801 or bevacizumab predominantly blocks G0/G1 phase, and in ovarian cancer, G2/M phase.^16^, ^17^, ^18^ The results of the present study showed that the anlotinib group and the combined group predominantly blocked G2/M‐phase cells with 15.81 ± 2.08% and 66.56 ± 3.01)%, and the treatment protocol with the participation of anlotinib could increase the proportion of G2/M‐phase cells, while the proportion of S‐phase cells was slightly higher in the radiotherapy group. This indicates that anlotinib blocks H520 cells in the G2/M phase. In the cell cycle, the G2/M phase is the most sensitive to ionizing radiation, the G1 phase is relatively sensitive, and the S phase is radio‐resistant. The response of tumor cells to ionizing radiation is dependent on their cell cycles. At the same time, radiotherapy promotes apoptosis and enhances the efficacy of radiotherapy by affecting signaling pathways through intracellular DNA damage. Hence theoretically, anlotinib could increase the radiosensitivity of H520 cells. The significance of this synergistic effect is to reduce the drug dosage and prolong the duration of drug action, thus reducing the associated side effects.

For the blocking effect of anlotinib on the tumor cell cycle, it was validated in this study by detecting the levels of CDK1 and cycle B affecting the G2/M phase. For the blocking effect of anlotinib on the tumor cell cycle, it was validated in this study by detecting the levels of CDK1 and cycle B which affect the G2/M phase. The CDK1 and cycle B expression levels were found to be significantly higher in the anlotinib group than in the control group. The key to blocking cells in the G2/M phase is to maintain the stability of maturation promoting factor (MPF), which consists of CDK1 and cycle B. CDK1 activity is regulated by the phosphorylation of Myt1 and Wee1. Wee1 is a tyrosine kinase located in the nucleus,^19^, ^20^, ^21^ and the biological effect of Wee1 is diminished by anlotinib, which decreases CDK1 activity, thus maintaining the structural stability of the MPF. Myt1 is a serine/threonine kinase that is itself regulated by anlotinib. The accumulation of CDK1 and cycle B in the present study in the anlotinib group indicates that MPF is in a relatively stable state. Tumor cells stagnated in G2 phase, on the one hand, inhibited tumor proliferation and on the other caused cells to accumulate DNA damage to induce apoptosis, and this result was confirmed in the experiment. Meanwhile, we also found that radiotherapy had an extremely strong killing effect on cells, and the expression levels of CDK1 and cycle B were lower in the radiotherapy‐only group than in the anlotenib group. However, in the anlotinib + radiotherapy group, CDK1 and cycle B expression levels were again lower than those in anlotinib‐only group due to the addition of radiotherapy, which was probably related to a higher proportion of apoptosis.

To study in depth the inhibition of cell cycle by the combination of anlotinib and radiotherapy, we attempted to investigate apoptotic cells in each group. It is known that, unlike cell necrosis, apoptosis performs programmed cell death by apoptotic signal transduction. Therefore, by studying the apoptosis signaling pathway, we can better understand the influence of interfering factors on the cell cycle. In this study, the expression of cysteine‐containing aspartic enzyme caspase was used to investigate the effects of anlotinib and radiotherapy on cell cycle. Caspase is a hemophenine protease, which is a key substrate in the signal transduction pathway of apoptosis. According to the role of caspase family members in the apoptosis pathway, they can be classified as initiator caspases and effector caspases. The effector caspase is directly involved in cutting the intracellular substrate and destroying the cell structure, which is the direct executor of cell apoptosis. Endogenous, exogenous and granzyme B pathways ultimately activate caspase‐3 as effector caspase. Therefore, the expression of caspase‐3 can represent the apoptosis of cells. In this study, we detected the expression of caspase‐3 in each group and found that both anlotinib and radiotherapy could significantly improve the apoptosis of tumor cells, and the combination of both could significantly enhance apoptosis of tumor cells. Radiotherapy can inhibit tumor cells mainly by promoting apoptosis and inducing autophagy. As a multitarget tyrosine kinase inhibitor targeting VEGFR, PDGFR, FGFR and C‐KIT, anlotinib promotes apoptosis by regulating the C‐Kit /PI3K/AKT pathway. Anlotinib and radiotherapy share some similar biological signal transduction pathways, thus providing a theoretical basis for their synergistic effect.

In conclusion, the radiation sensitization effect of anlotinib on H520 cell line is mainly reflected in two aspects: in the cell cycle, anlotinib blocks tumor cells to stay in the G2/M phase, creating conditions for the implementation of radiotherapy; at the cell growth block level, radiotherapy can work synergistically with anlotinib to block cell growth more effectively. Current clinical reports on the use of anlotinib for third‐line treatment of SqCLC^22^ are lacking large‐scale clinical data, but the available results are still impressive. In this study, in vitro experiments confirmed the higher tumor cell inhibitory capacity of the combination of anlotinib and radiotherapy, and confirmed the above clinical findings. However, due to the presence of more confounding factors, drug interactions, and radiation resistance in in vivo experiments, there is more uncertainty about the clinical use of anlotinib. In addition, there is a lack of data to quantify the synergistic effects of anlotinib and radiotherapy, and the relationship between the dosages of radiotherapy and anlotinib remains to be studied.
